# Cultivated land transformation and new-type urbanization couple from Imbalance to coordination in China’s Fenhe River Basin

**DOI:** 10.1038/s41598-026-54118-7

**Published:** 2026-05-25

**Authors:** Jianxin Fu, Min Jiang, Zhiping Wu, Maya Han, Wenbang Gao, Geng Liu

**Affiliations:** 1 Institute of Urban and Regional Development, Taiyuan Normal University, Jinzhong, 030619 China; 2 School of Geographic Sciences, Taiyuan Normal University, Jinzhong, 030619 China; 3 Shanxi Key Laboratory of Earth Surface Processes and Resource Ecology Security in Fenhe River Basin, Taiyuan Normal University, Jinzhong, 030619 China

**Keywords:** Transformation of cultivated land use, New-type urbanization at the county level, Coupling coordination degree model, Fenhe river basin, Influencing factor, Environmental impact, Agroecology, Urban ecology, Socioeconomic scenarios, Sustainability

## Abstract

This study examines the complex interdependent relationship between the transformation of cultivated land use and new-type urbanization at the county level within the Fenhe River Basin, highlighting its significant practical implications for the sustainable use of cultivated land and the advancement of high-quality new-type urbanization at the county level in China. Utilizing methodologies such as kernel density estimation, the coupling coordination degree model, and geographic detector methods, this research assesses the comprehensive levels of both cultivated land use transformation and new-type urbanization at the county level. Additionally, it explores the coupling relationship between these two phenomena and identifies their influencing factors. The findings indicate that:(1)There has been a consistent increase in the level of cultivated land use transformation, accompanied by a notable rise in the number of counties reaching high transformation level.(2)The comprehensive level of new-type urbanization at the county level has also shown a steady increase, with the proportion of counties at very high urbanization levels climbing to 43.9%, and these counties exhibit a clustered spatial distribution.(3)The overall level of coupling coordination between cultivated land use transformation and new-type urbanization at the county level has improved, suggesting enhanced coordinated development and reduced contradictions between these two processes. Notably, 90.24% of counties have upgraded their coupling coordination degree to either intermediate or high-levels.(4)The spatial differentiation of coupling coordination levels between cultivated land use transformation and new-type urbanization at the county level in the Fenhe River Basin has evolved from intermediate dissonance to high-level coordination, with this spatial pattern associated with a combination of natural and socio-economic factors. These insights will support the orderly utilization of cultivated land resources and foster the high-quality development of county-level urbanization in the Fenhe River Basin.

**Supplementary Information:**

The online version contains supplementary material available at 10.1038/s41598-026-54118-7.

## Introduction

Urbanization is a pivotal force driving social and economic progress, serving as a crucial pathway to achieving national modernization^1^. By the end of 2023, the urbanization rate of permanent residents in China had reached 66.16 percent. In light of the profound transformations in both domestic and international environments and conditions, urbanization must transition into a new phase of development that prioritizes quality enhancement^2^. Moving forward, we are committed to steadily advancing the high-quality development of people-oriented new urbanization that embodies Chinese characteristics. Urbanization supports the ongoing transfer and concentration of rural surplus labor as well as secondary and tertiary industries into urban areas. This process not only bolsters the regional industrial structure but also transforms the regional land use pattern^3^. The three control lines—the ecological conservation red line^4^, permanent basic farmland, and urban development boundary—constitute non-negotiable spatial governance boundaries that jointly safeguard ecological security, ensure national food security, and guide the orderly, sustainable expansion of urbanization. Rural areas are the fundamental material foundation and spatial carrier for the development of new-type urbanization at the county level in China. As new-type urbanization at the county level rapidly progresses, especially in peripheral regions, these areas face numerous challenges. These include the degradation in both the quantity and quality of arable land^5^, threats to food security^6^, and barriers to urban-rural integration^7^ caused by urban expansion. The encroachment of urban construction on cultivated land leads to a disruption in the spatial development order^8^, inefficient and extensive land use^9^, and a misalignment between the transformation of cultivated land use and the development of new-type urbanization at the county level. To proactively guide the transformation of cultivated land use and the development of new-type urbanization at the county level towards a synergistic relationship, it is essential to clarify the coupling and coordinated development mechanisms between the two. Investigating the factors influencing this coupling relationship is of significant practical importance for aligning the transformation of cultivated land utilization with the development of new-type urbanization at the county level.

The primary research findings concerning the transformation of cultivated land use and the development of new-type urbanization at the county level can be summarized as follows: (1) The transformation of cultivated land use focuses on three main areas: the spatial dynamics of changes in cultivated land^10^, the sustainable transition of cultivated land use^11^, and the spatio-temporal characteristics of these changes along with their driving factors^12^. Research in this field explores various perspectives including the effects on carbon emissions^13^, carbon storage^14^, and policies aimed at the protection of cultivated land^15^. Zhang Panpan et al. carried out a quantitative analysis of the spatio-temporal differentiation of cultivated land in the Weibei arid plateau region of China, offering a detailed examination of the mechanisms driving these changes^16^. Furthermore, Cheng Hui et al. employed GIS technology to study the spatio-temporal characteristics of cultivated land resources, focusing on aspects such as the total amount, the degree of development and utilization, the rate of dynamic change, and the central trends and directions of usage^17^. Their research aimed to provide a scientific basis for the rational development, utilization, and protection of cultivated land resources in China. Various scholars have explored the coupling relationship between the transformation of cultivated land use and ecosystem service value^18^, as well as changes in the rural labor force^19^ and their impact on farmer income^20^ from multiple perspectives.(2) New-type urbanization at the county level: This topic primarily deals with quality assessment^21^, dynamic mechanisms^22^, spatio-temporal evolution^23^, and development models^24^. Numerous researchers have investigated the connections between new-type urbanization at the county level and various developmental dimensions, including economic spatial development^25^, urban resilience^26^, ecosystem services^27^, carbon emissions^28^, and food production^29^. The research on the coupling relationship between the transformation of cultivated land use and new-type urbanization at the county level, along with its influencing factors, requires further enhancement and deeper investigation.

Although the coupling and coordination between the transformation of cultivated land utilization and New-type urbanization at the county level had attracted scholarly attention, existing studies remain limited in two critical aspects: first, they inadequately elucidate the specific transmission pathways and mediating mechanisms underlying this coupling; second, they predominantly treat coupling coordination as a technical tool for quantitative modeling—without sufficiently grounding it in conceptual clarity or theoretical rigor. Consequently, the substantive, bidirectional interactions between land-use change and urbanization processes remain undertheorized. To address these gaps, this study develops a comprehensive theoretical framework that explicates the structural logic, causal mechanisms, and contextual conditions shaping their coupling and coordination—thereby advancing both conceptual understanding and policy-relevant insights for synergistic cultivated land protection and high-quality urbanization.

**Fig. 1:**
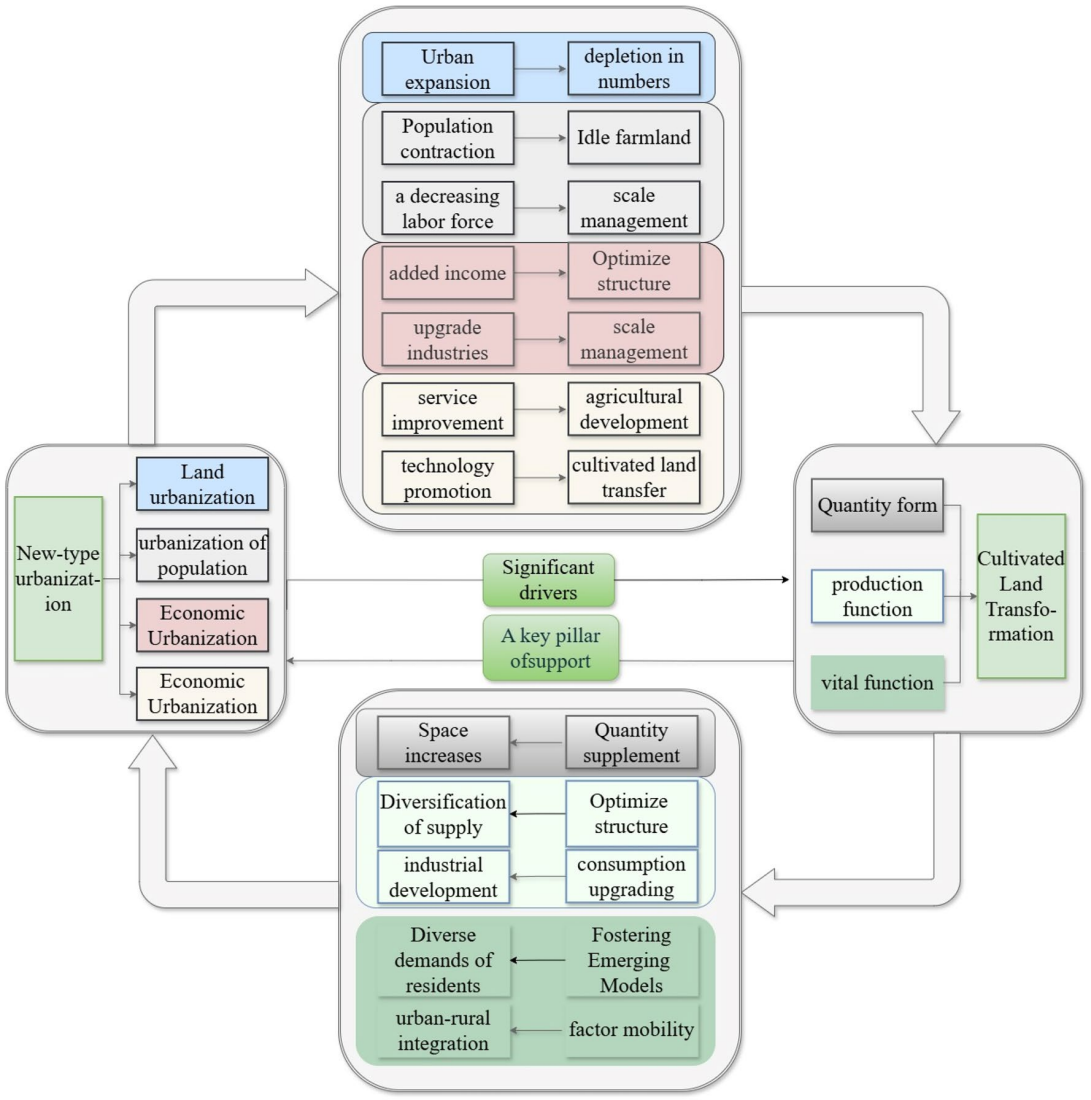
The coupling mechanism between cultivated land transformation and new-type urbanization at the county level.

The coupling relationship between the transformation of cultivated land utilization and New-type urbanization at the county level is essentially characterized by the flow and integration of production factors between the cultivated land utilization system and the county-level urbanization system(see Fig. 1). This interaction forms a complex coupling relationship where both systems influence and shape each other. The transformation of cultivated land utilization serves as the critical foundation for the New-type urbanization at the county level. Its impacts on the New-type urbanization at the county level encompass various dimensions, including quantitative aspects, production functions, and living functions. (1) Quantity-based pattern : In order to enhance the management of territorial space resources, during the implementation of policies such as balancing cultivated land occupation^30^ and compensation and linking the increase and decrease of construction land, the government supplements a significant amount of cultivated land by integrating various types of land consolidation projects through comprehensive planning. Not only does it enable large-scale operation of cultivated land, but it also releases more land resources for urbanization construction, thereby facilitating the advancement of urbanization in counties to a new level. (2) Production function: As the fragmentation and non-grain utilization of cultivated land accelerate, the planting structure of cultivated land^31^ has undergone continuous optimization, thereby diversifying the assurance of agricultural product supply. At the same time, the upgrading of residents’ consumption demands has not only driven the development of industries such as the deep processing of agricultural products and rural tourism but also facilitated the enhancement of the urbanization level within the county economy. (3) Living functions: The living functions of cultivated land have given rise to new business models, transitioning cultivated land from a singular focus on grain production to encompassing multiple and composite functions such as leisure, sightseeing, and experiential activities. This transformation fulfills the varied needs of urban residents. The transformation of the living functions of cultivated land has facilitated the accelerated flow of factors such as capital, technology, and consumption between urban and rural areas, promoted the development of infrastructure including roads, networks, and public services, reduced the urban-rural gap, and advanced the integration of urban and rural areas. The New-type urbanization at the county level plays a crucial role in facilitating the transformation of cultivated land utilization through the interrelated dimensions of land urbanization, population urbanization, economic urbanization, and social urbanization. (1) Land Urbanization: Driven by the relatively low cost of converting arable land to non-agricultural uses, the rapid expansion of urban land has significantly accelerated the depletion of arable land resources. This process has directly or indirectly led to changes in both the quantity and spatial distribution of arable land. (2) Population Urbanization: Driven by disparities in factors such as income, education, and healthcare between urban and rural areas, the rural population has significantly declined^32^, leading to the underutilization or non-agricultural conversion of farmland. Meanwhile, the decline in the agricultural labor force has driven an increase in the level of mechanization, thereby facilitating the consolidation of farmland and promoting large-scale operations. (3)Economic urbanization: The growth of the non-agricultural economy has resulted in higher household incomes and a shift towards more diversified food consumption patterns, thereby facilitating the conversion of farmland into areas dedicated to high-value economic crops. The upgrading of the industrial structure and the pull effect of non-agricultural employment opportunities have driven the expansion of large-scale cultivated land operations. Meanwhile, due to the substantial economic benefits associated with the non-agricultural use of cultivated land, the rate of change in the transfer of cultivated land use in suburban areas has accelerated. (4)Social urbanization: The enhancement of public service levels, including education and healthcare, contributes to improving the quality and well-being of the labor force. The transformation of business concepts has further accelerated the promotion of new agricultural technologies^33^, fostering the development of advanced agriculture types such as smart agriculture. This has not only improved agricultural labor efficiency but also enhanced farmers’ willingness to transfer cultivated land and operate on a larger scale.

The Fenhe River Basin, located in Shanxi Province, is a critical ecological functional area known for its dense population, significant agricultural production (primarily grain and cotton), economic advancement, and urban agglomeration. However, the rapid urbanization at the county level has led to problems such as unchecked expansion of construction land^34^ and disorganized management of cultivated land resources. As of 2022, the urbanization rate of permanent residents in the Fenhe River Basin was 67.96%, which exceeds the national average by 1.8 percentage points. Between 1990 and 2020, the proportion of cultivated land in the total area of the basin decreased from 41.52% to 39.23%. During this period, issues including the marginalization of farmland, non-agricultural use, fragmentation^35^, abandonment, and degradation of land quality have become increasingly significant. The escalating conflict between the transformation of cultivated land use and the development of county-level urbanization in the Fenhe River Basin has become a major challenge, significantly impacting both the sustainable use of farmland and the high-quality development of urbanization at the county level. This study focuses on the Fenhe River Basin and develops an index system to analyze the transformation of cultivated land utilization and the development of county-level urbanization. Utilizing mathematical methods and models, such as the coupling coordination degree model and geographic detector, this study explores the coupling relationship between these two processes and their influencing factors. The objective is to provide guidance for the rational transformation and utilization of cultivated land resources within the basin, as well as to support the high-quality development of county-level urbanization.

## Materials and methods

### Measurement methods for cultivated land use transformation and new-type urbanization at the county level

Drawing on relevant research^36^ and considering the specific conditions of the study area, an index system was constructed to assess the transformation of cultivated land use and urbanization at the county level, adhering to principles of scientific rigor, comprehensiveness, and practicality^37^. The weights of the indices were determined using the entropy method to evaluate the overall level of transformation and urbanization.

#### Holistic assessment of cultivated land use transformation

Based on prior research^38,39^ and considering the unique aspects of cultivated land use transformation in this region, an index system was developed to measure its comprehensive level. This system includes three dimensions: quantitative metrics, production functionality, and life support function, as outlined in Table 1. The quantitative aspect of cultivated land reflects the changes in its area or quantity over time and space, while the production function measures its capability to produce agricultural outputs. Furthermore, the life support function emphasizes its role in providing livelihood security for farmers and ensuring food security^40^. In this study, the per capita area of cultivated land is used to represent both the quantity and spatial distribution of cultivated land. The multiple cropping index, grain yield per unit area, and the seeding ratio of grain crops are used to quantify its production function. Moreover, the per capita grain guarantee amount and the total power of agricultural machinery are utilized to illustrate the life support function of cultivated land.

**Table 1:** Evaluation index system for assessing the comprehensive level of cultivated land use transformation.

Target layer	Criterion layer	Indicator layer	Calculation method	The rationale for index selection	Attribute	Weight
Holistic Assessment of Cultivated Land Use Transformation	Quantitative form	Per capita cultivated area (hm^2^/person)	Arable land area per capita in the region	It indicates the abundance and per capita availability of regional cultivated land resources.	﹢	0.2679
	Production function	cropping index(%)	Total sown area or cultivated area of crops	It indicates the efficiency of cultivated land use and the intensity of agricultural production.	﹢	0.1119
		Grain Crop Seeding Ratio (%)	Cultivated area for food crops / total cultivated area	It can reflect regional food self-sufficiency and the agricultural structure.	﹢	0.0507
		Grain yield per unit area (t/hm^2^)	Total grain yield per unit area of sown grain crops	It serves as a clear indicator of advancements in agricultural technology.	﹢	0.1813
	Life function	Per capita food security (t/person)	Food production per capita in the region	It serves as an indicator of the region’s food supply capacity.	﹢	0.3269
		Total agricultural machinery power per capita (kW/person)	Total power of agricultural machinery per capita in the region	It serves as an indicator of the level of regional agricultural mechanization.	-	0.0613

#### Holistic assessment of new-type urbanization at the county level levels

In response to the accelerating integration of urban and rural areas, the concept of urbanization has expanded to encompass a multifaceted array of dimensions. This evolution is reflected in the transition from reliance on simplistic population-based metrics to a more comprehensive evaluation index system, which now includes economic, social, land use, demographic, and regional landscape indicators. Based on the synthesis of prior studies^41–43^, a novel index system was established to assess the comprehensive urbanization level at the county scale. This system integrates four key dimensions—land, population, economy, and society—and is composed of specific indicators such as land use efficiency and urban population density (Table 2). Altogether, the system features ten indicators aimed at providing a quantitative measurement of the overall urbanization level in counties.

**Table 2:** Evaluation index system for the comprehensive level of new-type urbanization at the county level.

Target layer	Criterion layer	Indicator layer	Calculation method	Reasons for selecting this indicator	Attribute	Weight
Holistic sssessment of new-type Urbanization at the county level Levels	Urban land expansion the urbanization of the population	Construction land percentage (%)	Built-up area / Total land area	It serves as an indicator of the intensity of land development during the urbanization process.	﹢	0.0640
		Land use efficiency (10^4^ yuan/km^2^)	GDP per unit of construction land area	It indicates the economic output per unit area of land.	﹢	0.1111
		Urban population		It effectively demonstrates the population density of the city.	﹢	0.0911
		Permanent resident urbanization rate (%)	Urban permanent population as a proportion of total population	It serves as the core indicator for measuring the level of population urbanization.	﹢	0.0376
		Urban population density (persons per km^2^)	Urban population/total area	It serves as a crucial indicator for measuring the level of population urbanization.	﹢	0.2338
	Economic Urbanization	GDP (per ten thousand yuan)		It serves as an indicator of the city’s economic scale and overall development level.	﹢	0.1306
		Added value of the secondary industry (in Ten Thousand Yuan)		It effectively reveals the economic structure and level of industrialization in the city.	﹢	0.1202
		GDP per capita (in Yuan)	GDP/total population	It serves as an indicator of the average income level and spending power of residents.	﹢	0.0860
	Social Urbanization	Total student population in the school		It can reflect the city’s level of educational development and public service capacity.	﹢	0.0310
		Number of beds in hospitals and health centers		It serves as a reflection of the city’s medical resources and service capabilities.	﹢	0.0946

### Kernel density

The method of kernel density estimation^44^ was utilized to analyze the patterns of coupling and coordinated changes between cultivated land use transformation and new-type urbanization at the county level in the Fenhe River Basin. The formulation of this method is as follows:

1 $${\mathrm{f}}_{k} = \frac{1}{Nh}\sum\limits_{i = 1}^{N} {K\left( {\frac{{X_{i} - x}}{h}} \right)}$$

In this equation, N indicates the number of observed values; h is the bandwidth; X_i_ denotes the ith observed value;x is the mean value; and K(·) represents the kernel function, which is calculated using the Gaussian function. The height of the kernel density curve reflects the degree of agglomeration in the coupling coordination among counties, whereas the curve’s width illustrates the dispersion of these degrees. The number of peaks indicates the polarization in coupling coordination degrees, and the distribution ductility shows the range between the highest and lowest degrees across counties. The tail of the curve emphasizes significant spatial disparities in the coupling coordination degrees.

### Model of coupling coordination degree

The coupling degree (C) is a vital metric for quantifying the interaction level between different systems or elements. The Integrated Coordination Index (T) captures the overall synergy and performance of these systems. Building on this foundation, the coupling coordination degree model (D) was employed to quantify the interrelation between cultivated land use transformation and new-type urbanization at the county level^45^. The formulas are as follows:

2 $$C = \left\{ {u_{1i} \times u_{2i} /\left[ {\left( {u_{1i} + u_{2i} } \right)/2} \right]^{2} } \right\}^{1/2}$$

3 $$T = W_{1} u_{1i} + W_{2} u_{2i}$$

4 $$D = \sqrt {C \times T}$$

Within these formulas, C represents the coupling degree; T indicates the comprehensive coordination index; D denotes the coupling coordination degree; u_1i_ and u_2i_ are the measurement indices for cultivated land use transformation and new-type urbanization at the county level, respectively. Given that both indicators are equally critical and functionally interdependent within the regional sustainable development system—and given the absence of a universally accepted, objective weighting methodology—equal weighting is adopted to minimize subjective judgment, enhance result robustness and cross-study comparability, and align with established scholarly practice^46^. W_1_ and W_2_ are the weighting coefficients for these indices, both set at 0.5 in this study. Based on the contextual review of relevant literature^46^, the coupling coordination degree is stratified into five categories: primary dissonance(0.3-0.4), intermediate dissonance(0.4-0.5), primary coordination(0.5-0.6), intermediate coordination (0.6-0.7), and high-level coordination (>0.7).

### Geographic detector

The Geographic Detector^47^ is a statistical tool developed to ascertain the determinants of spatial heterogeneity in various geographic elements and phenomena. This method comprises factor detection and interaction detection, which facilitate the analysis of spatial variations in univariate data and the exploration of potential causal relationships in bivariate data. The mathematical representation employed is as follows:

5 $$q = 1 - \frac{{\sum\limits_{h = 1}^{L} {N_{h} \sigma_{h}^{2} } }}{{N\sigma^{{2}} }}$$

In the equation, q represents the influence factor; N_h_ and N denote the number of units in layer h (where h = 1, 2, ..., L) and the total sample size of the study area, respectively; σ_h_^2^ and σ^2^ symbolize the variances of layer h and the overall study area Y values, respectively. The value of q ranges from 0 to 1, where a higher q value indicates a stronger explanatory power of the factor on the interaction and coordinated progress between cultivated land use transformation and new-type urbanization at the county level. Conversely, a lower q value suggests a weaker explanatory influence. Specifically, q=1 denotes the strongest explanatory power, while q=0 implies no association with the geographical phenomena.

### Overview of the study area

**Fig. 2:**
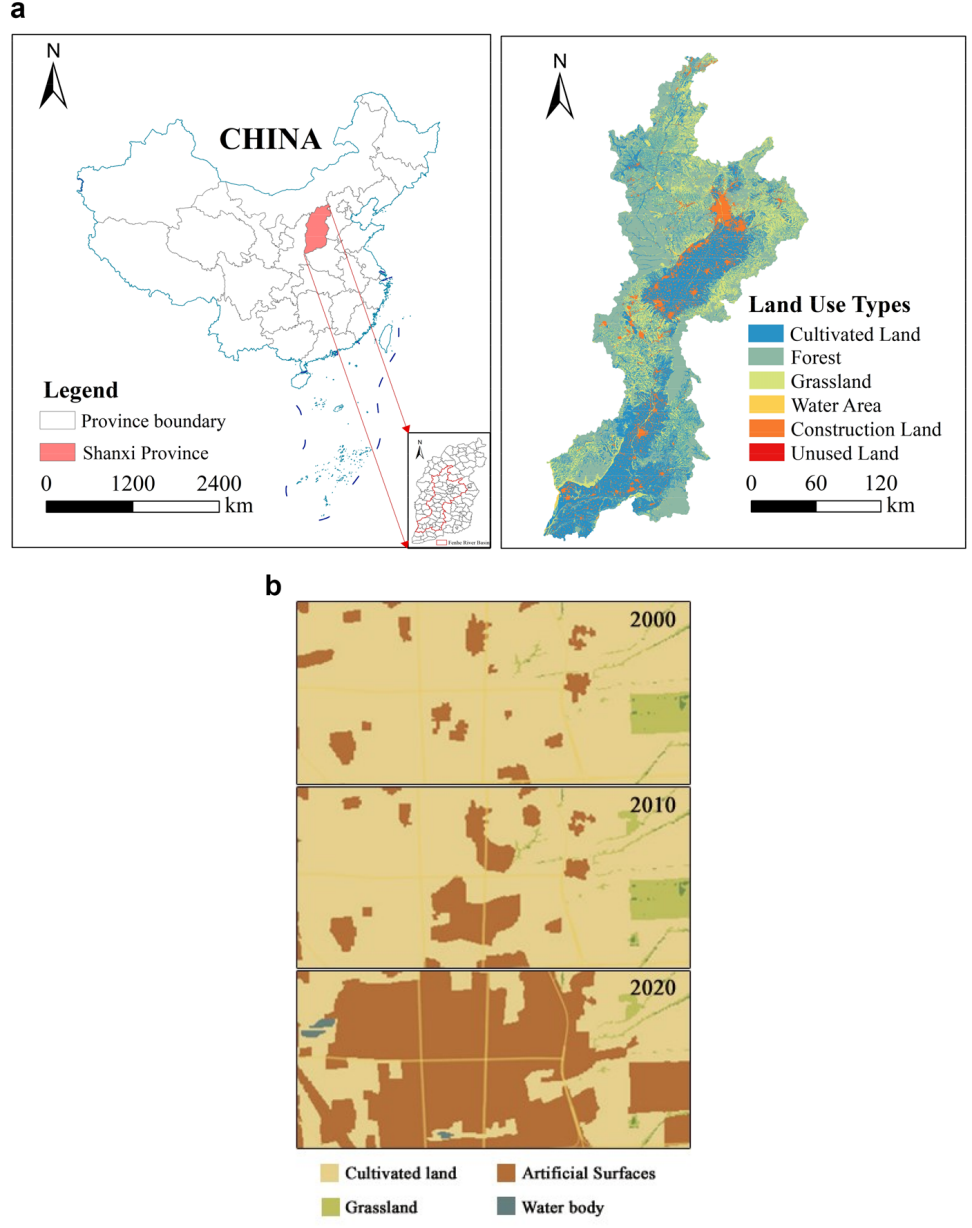
(**a**) Overview of the study area. *Note*: The base map was created using the standard map of the Ministry of Natural Resources, with the map review number GS(2016)1609. The boundaries of the base map have not been modified in any way. The same applies below. In addition, the administrative boundaries of the Fenhe River Basin in 2020 were adopted in this article. It was made by ArcGIS 10.8 https://www.esri.com/en-us/arcgis/products/arcgis-online/overview. (**b**) Comparison chart of different land categories during the process of urbanization. The original data is sourced from the National Platform for Common GeoSpatial Information Services https://cloudcenter.tianditu.gov.cn/landCover. It was made by Adobe Photoshop CS3 https://www.adobe.com/products/photoshop.html.

The Fenhe River, the second-largest tributary of the Yellow River, flows through the ecologically sensitive Loess Plateau region, situated in the middle reaches of the Yellow River^48^. The river’s basin extends longitudinally from 110°26’E to 113°37’E and latitudinally from 35°05’N to 39°14’N, covering approximately 25% of Shanxi Province’s total area. Elevations within the basin range from 351 to 2799 meters above sea level, as depicted in Fig. 2a. The basin is flanked by the Lvliang Mountains to the west and the Taiyue and Zhongtiao Mountains to the east, and includes the Jinzhong and Linfen Basins in its central region. The area is characterized by a temperate continental monsoon climate, with rainfall and high temperatures typically occurring simultaneously. There are significant fluctuations in temperature, both daily and annually, and precipitation is unevenly distributed across different seasons and years. The study area includes 41 counties. Given the extensive duration of the research and the significant area of cultivated land within these municipal districts, they have been included in the study area. Urbanization at the county level has led to a continuous expansion of urban areas, resulting in significant encroachment of construction land on cultivated land at the urban-rural interface.

In 2022, the permanent resident population of the Fenhe River Basin was approximately 15.2406 million. The output values of the secondary and tertiary industries represented 40.52% and 49.40% of the provincial totals, respectively. The per capita disposable income of residents was 28,200 yuan, 2,700 yuan more than that of the residents in Shanxi Province. Total retail sales of consumer goods in rural areas amounted to 47.477 billion yuan, accounting for only 14.05% of the basin’s total retail sales. The Fenhe River Basin acts as a vital ecological buffer zone, playing a crucial role in maintaining the ecological security of the Yellow River and serving as a key focus of national ecological conservation efforts. Shanxi Province launched a farmland-to-forest and grassland conversion project in 2000. Over two decades, this initiative successfully converted approximately 27.303 million mu of farmland into forested and grassland areas, leading to a noticeable improvement in the ecological environment of the basin. From 1990 to 2020, the urban land area within the basin expanded from 35,013.45 hectares to 94,300.38 hectares, an increase of 169.33%. The proportion of urban land relative to the total area designated for construction rose from 22.55% to 29.14%. Taking 2000, 2010 and 2020 as three time periods, the University Town in Yuci County, Jinzhong City was selected as a typical case study area to compare the changes in land types such as cultivated land and urban land during its urbanization process (Fig. 2b).In this context, ensuring the rational transformation of cultivated land use and promoting high-quality urbanization of counties are crucial for future urban development and rural revitalization in the basin.

### Data sources

For analyzing land cover changes in the years 1990, 2000, and 2010, Landsat TM/ETM+ remote sensing image data were utilized, while Landsat 8 OLI remote sensing image data were employed for the 2020 analysis. The overall accuracy of these interpretations exceeded 95%. Socioeconomic data were sourced from various publications including the Shanxi Provincial Statistical Yearbook, China County Statistical Yearbook, Taiyuan Statistical Yearbook, and annual statistical bulletins on national economic and social development issued by each county. Missing data points were filled using interpolation techniques. To ensure comparability across years, the temporal consistency of all employed datasets, especially remote sensing data and socioeconomic data, was clearly maintained. All variables were collected for consistent time periods throughout the study. Digital Elevation Model (DEM) data were acquired from the Geospatial Data Cloud (https://www.gscloud.cn/). Additionally, temperature and precipitation data were collected from the China Meteorological Network(Table 3).

**Table 3:** Data sources in this study.

Data type	Sources
Land use/cover	Data Center for Resources and Environmental Sciences, Chinese Academy of Sciences (RESDC) (http://www.resdc.cn)
Digital elevation model	Geospatial data cloud(https:∥www.gscloud.cn/)
Socioeconomic data	Shanxi statistical yearbook, China statistical yearbook((County-level),Tai yuan statistical yearbook
Air temperature and precipitation data	The China meteorological data service center(http://data.cma.cn/)

## Results

### Analysis of the integrated assessment of cultivated land use transformation and new-type urbanization at the county level

#### Holistic transformation of cultivated land utilization

**Fig. 3:**
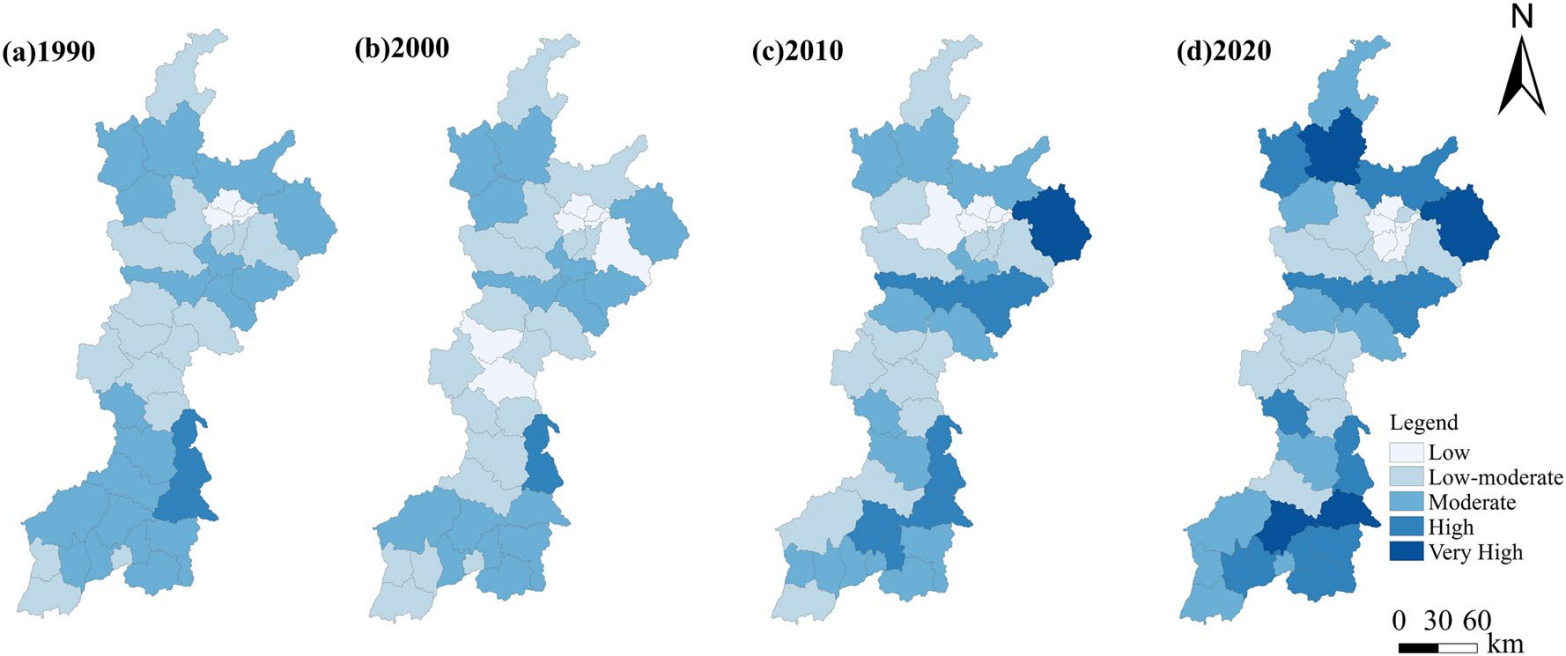
Spatial distribution of the comprehensive level of cultivated land use transformation. It was made by ArcGIS 10.8 https://www.esri.com/en-us/arcgis/products/arcgis-online/overview.

Drawing on previous studies^49^, ArcGIS 10.8 was employed to delineate the evolution of the comprehensive spatial dynamics of cultivated land use transformation(Fig. 3). This transformation process was stratified into five categories: < 0.2 (low), 0.2-0.3 (low-moderate), 0.3-0.4 (moderate), 0.4-0.5 (high), and > 0.5 (very high).

From 1990 to 2020, the Fenhe River Basin witnessed a fluctuating yet generally upward trend in the level of cultivated land use transformation, with significant spatial variations presenting higher levels in the northern and southern regions compared to the central area.

In 1990, the Fenhe River Basin’s overall level of cultivated land use transformation was not high. Xinghualing County, Jiancaoping County and two other counties belong to the low level area, accounting for 9.76% of the total number of counties. 39.02% of counties were categorized at low–moderate level, these areas were predominantly located in the urban sectors of Taiyuan City and the middle reaches of the river basin, presenting a scattered and clustered distribution. The high level regions were limited to Gu County and Fushan County.

By 2000, the proportion of counties at a low level had increased to 17.07%, displaying a sporadic distribution. The count of counties at the low–moderate level rose to 18, showing a clustered pattern. Conversely, the number of counties at the moderate level decreased slightly, with their proportion falling to 36.59%. The high level counties were exclusively Gu County. Overall, the comprehensive level of cultivated land use transformation in the basin demonstrated a declining trend, consistent with the "U-shaped" model of cultivated land use transformation^50^.

In 2010, there was an observable increase in the rate of cultivated land use transformation, with a spatial distribution pattern that showed higher levels in the southern regions and lower levels in the northern areas. Counties with low level of transformation were geographically clustered. The count of these low–moderate level counties decreased to 14, while moderate level counties were primarily found in Jingle County, Hongdong County, and Hejin City, displaying a distribution pattern that was both circular and dispersed. The number of counties classified at high level expanded from one to seven.

By 2020, the count of low-level counties remained stable. However, both Gujiao City and Xinghualing County advanced from a low to a low–moderate level, reducing the total number of low–moderate counties to 11. Xiangfen County and Fushan County both rose to the very high level category, thereby increasing the proportion of very high level counties from 2.44% to 9.76%. Additionally, seven counties, including Yangqu County, Lan County, and Fenxi County, ascended from the moderate level to the very high level. This elevation increased the proportion of high level counties to 29.27%, with a prominent spatial expansion towards the southeastern part of the basin. In summary, the number of counties at high and very high levels saw an increase, while those at low–moderate and moderate levels experienced a decrease. The number of counties at low levels remained largely consistent, underscoring a spatial distribution that featured higher levels in the southeastern region and lower levels in the northwestern region. The overall level of transformation of cultivated land utilization in the urban area of Taiyuan had always been relatively low, mainly due to its large population and small per capita land area.

#### Holistic assessment of new-type urbanization at the county level

**Fig. 4:**
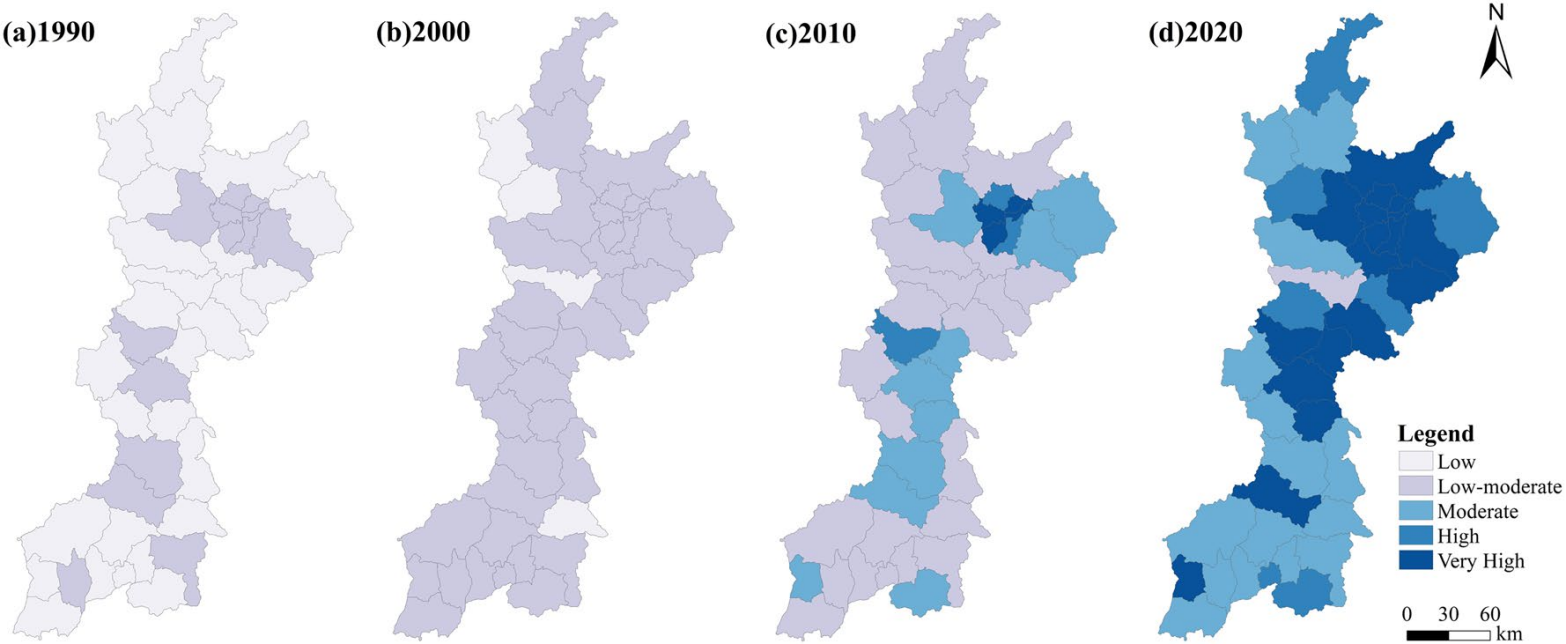
Spatial distribution pattern of county-level urbanization index. It was made by ArcGIS 10.8 https://www.esri.com/en-us/arcgis/products/arcgis-online/overview.

Drawing on prior research^51^ and the unique characteristics of the Fenhe River Basin, the comprehensive urbanization level of counties has been classified into five categories: low, low-moderate, moderate, high, very high (Fig. 4).

This framework facilitates a nuanced understanding of regional urbanization dynamics over a thirty-year period, from 1990 to 2020. The data indicate a consistent upward trajectory in urbanization across the basin during this period. Specifically, the average urbanization level rose from 14.73% in 1990 to 49.49% in 2020, marking a substantial overall growth rate of 235.98%. This was in line with the general trend of urbanization level improvement in the entire Yellow River Basin.

Initially, in 1990, 27 counties, including Huozhou City, Jiaocheng County, and Jingle County, were designated as low level urbanized areas, comprising 65.85% of the total. Low-moderate counties were primarily concentrated in 14 counties, with a notable clustering in the Taiyuan urban area.

From 2000 onwards, the average urbanization level modestly increased to 20.21%, reflecting a growth rate of 37.2%. Concurrently, the proportion of counties categorized at the low level diminished to 1%, while the count of low-moderate level counties rose to 37, suggesting a shift towards a more concentrated distribution of urbanization. By 2010, the average rate of urbanization had reached 29.13%, an increase of 44.31%. Notably, no counties were classified at the low level urbanization level by this point. Four counties, including Lan County and Loufan County, it upgraded from the low level category to the low-moderate level category and were more dispersed geographically. The low economic development levels of these counties have led to a relatively low level of urbanization. Ten counties, such as Gujiao City and Shouyang County, progressed from low-moderate to moderate level, accounting for 24.39% of the total. Xiaoyi City, Xianqiang County and Xiaodian County have risen from Low–moderate levels to high levels. Xiaoyi City mainly relied on its abundant natural resources to develop its economy, and the high-level economy has promoted the process of urbanization. Further, several counties within Taiyuan City, including Xinghualing County, Wanbailin County, Yingze County, and Jinyuan County, ascended from low-moderate to very high level, representing 9.76% of the total. Taiyuan City, as the provincial capital of Shanxi Province, had a significant influence on the urbanization levels of surrounding areas through its radiation and leading role. In 2020, the number of counties at the very high level had expanded from 4 to 18, increasing their share to 43.9%. Xiaoyi City, Jiancaoping County, and Xiaodian County notably advanced from the high to the very high category. Additionally, the number of high level counties reached 7. Jiang County and Shouyang County progressing from moderate to high level. The count of moderate level counties grew to 15, making up 36.59% of the total, while only Wenshui County remained at the low-moderate level.

### Spatio-temporal evolution of the coupling coordination degree between cultivated land use transformation and new-type urbanization at the county level

#### Temporal characteristics of coupling coordination levels

**Fig. 5:**
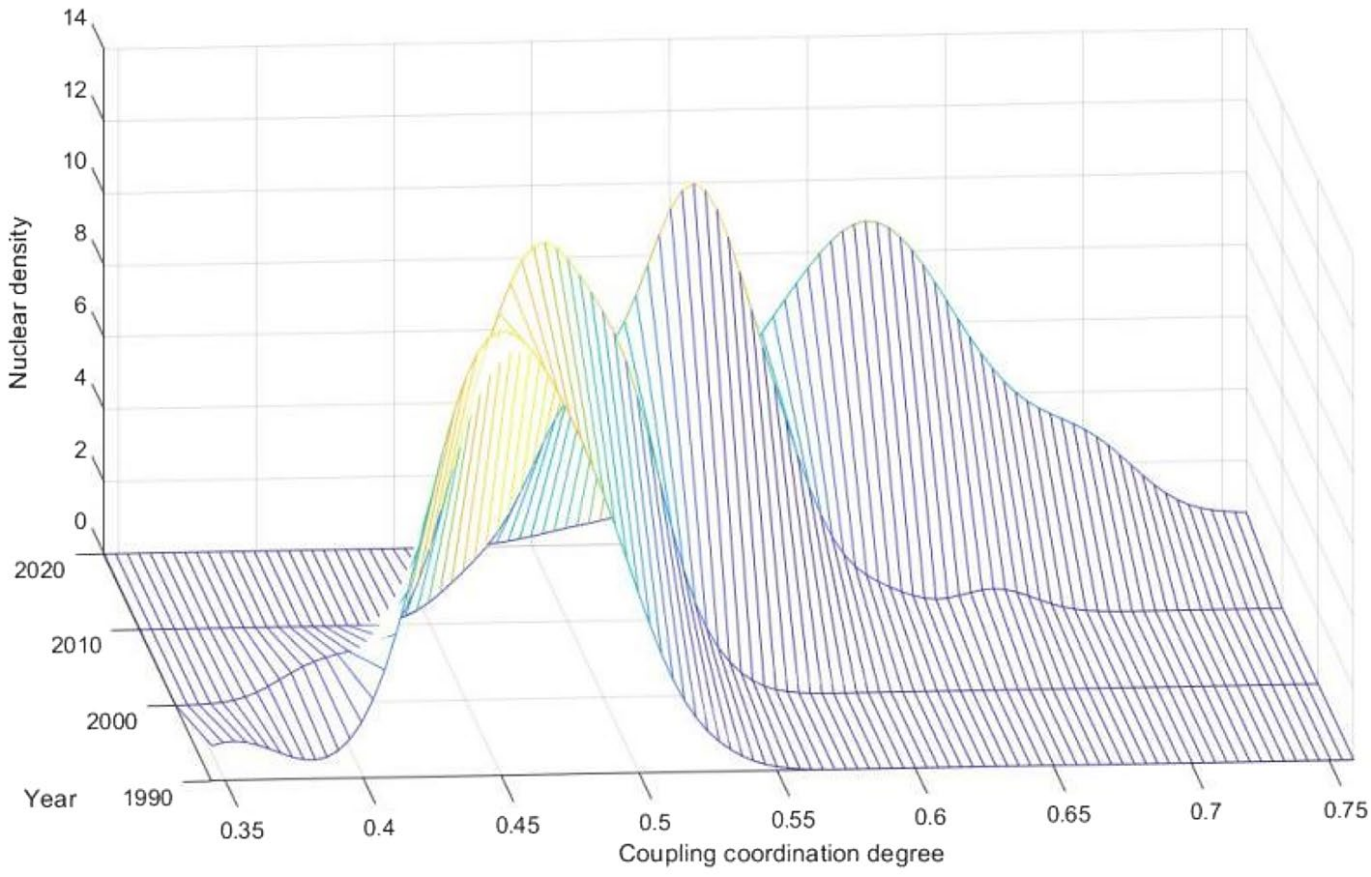
Temporal evolution of the coupling coordination degree between cultivated land transformation and new-type urbanization at the county level.

The kernel density estimation method was utilized to analyze the coupling coordination degree between the transformation of cultivated land use and urbanization at the county level within the Fenhe River Basin (refer to Fig. 5). Over the period from 1990 to 2020, the kernel density distribution curve for the coupling coordination degree between these two variables demonstrated a progressive rightward shift, indicating a general enhancement in the level of coupling coordination. This trend reflects an increase in the statistical synchronization and matching between the two systems, rather than a direct reduction in conflicts between human activities and land resources. Observing the primary peak distribution pattern, there was a notable increase in both the height and breadth of the peak, reflecting an ongoing improvement in the coupling coordination degree among the counties (and cities) within the basin. Despite these improvements, the analysis also reveals that regional disparities are widening. Focusing on tracer ductility, the elongation of the left tracer alongside the shortening of the right tracer indicates an increase in disparity within regions of low coupling coordination, while a decrease in disparity is observed within regions of high coordination. From 2010 to 2020, the kernel density curve displayed a double-peak phenomenon, characterized by multiple wave peaks, suggesting a minor polarization effect within the levels of coupling coordination.

#### Spatial characteristics of coupling coordination levels

**Fig. 6:**
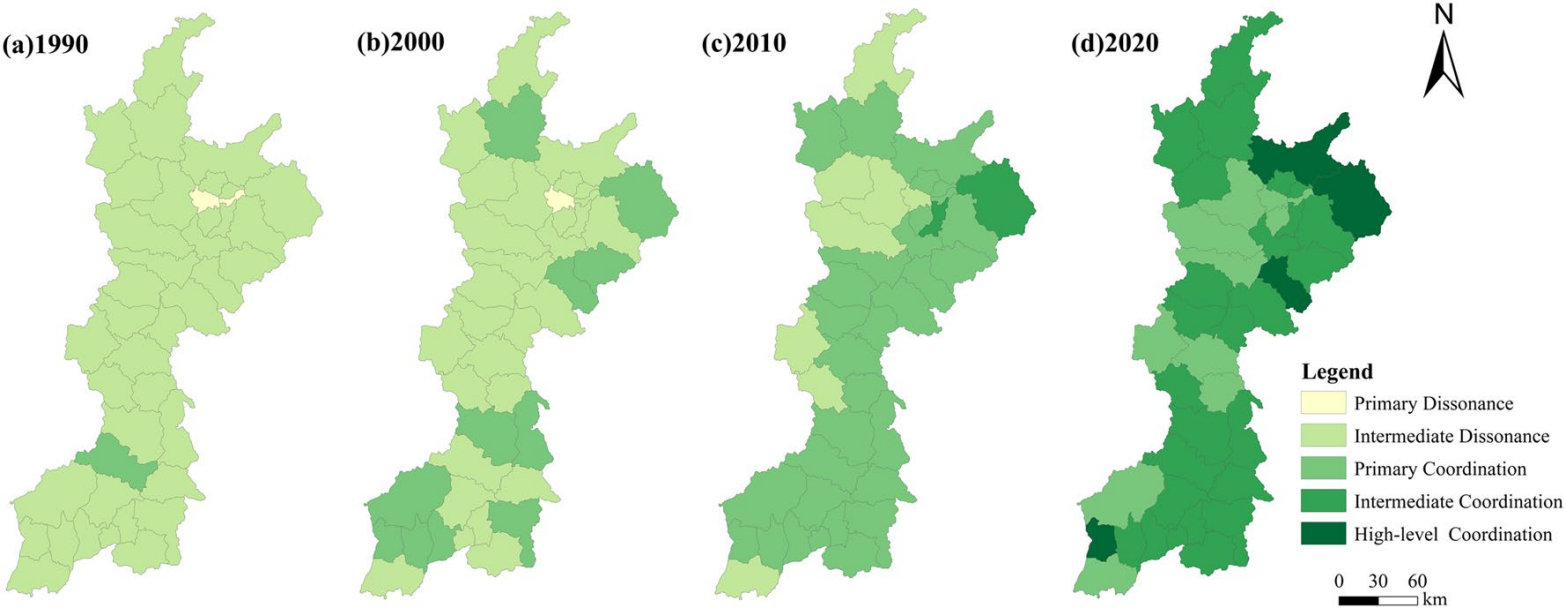
Spatial distribution pattern of the coupling coordination degree between cultivated land use transformation and new-type urbanization at the county level. It was made by ArcGIS 10.8 https://www.esri.com/en-us/arcgis/products/arcgis-online/overview.

From 1990 to 2020, the coupling coordination degree between cultivated land use transformation and new-type urbanization at the county level in the Fenhe River Basin demonstrated a spatial evolution trend towards balance and coordination, as depicted in Fig. 6.

In 1990, a substantial 92.68% of the counties within the Fenhe River Basin were categorized at the primary dissonance level. Notably, Wanbailin County and Yingze County in Taiyuan City were classified at the intermediate dissonance level, whereas Yaodu County in Linfen City was at the primary coordination level. By 2000, the overall coupling coordination degree of the Fenhe River Basin predominantly indicated an primary dissonance, with the proportion of counties experiencing this condition decreasing to 73.17%. Significantly, the area exhibiting primary coordination in the lower reaches of the basin expanded considerably, accounting for 26.83% of the counties and forming a clustered distribution. In the initial stage of urbanization, the expansion of urban infrastructure and residential areas had reduced both the area and quality of cultivated land. By 2010, the proportion of areas with primary coordination escalated to 80.49%, marking a significant rise of 53.66 percentage points. This shift signifies a considerable enhancement in coupling coordination levels and a reduction in the conflict between human activities and land use. During this period, 21 counties, including Fenyang City and Pingyao County, transitioned from the primary dissonance area to the primary coordination area, constituting 51.22% of the total number of counties. Additionally, Xiaodian County and Shouyang County advanced to an intermediate coordination region. In 2020, Shouyang County progressed from an intermediate coordination area to a high-level coordination area. This new high-level coordination area now includes Qi County, Hejin City, Yangqu County, and Shouyang County, representing 9.76% of the total area and exhibiting a point distribution pattern. Furthermore, 26 counties, including Gu County, Yicheng County, and Xinghualing County, ascended from primary coordination areas to intermediate coordination areas, thereby increasing the proportion of intermediate coordination counties to 63.41%. Consequently, 90.24% of the counties achieved either intermediate or high-level levels of coupling coordination, indicating a generally well-coordinated developmental status. With the implementation of energy conservation and emission reduction policies, the influence of macro policies on the transformation of cultivated land was gradually increasing, leading to a more coordinated development between the transformation of cultivated land utilization and urbanization.

Overall, the coupling coordination degree between farmland use transformation and new-type urbanization at the county level in the Fenhe River Basin has shown continuous improvement over the three decades. However, the number of counties achieving high-level coordination remains limited, highlighting the need for further enhancement.

### Geospatial analysis of impact factors on the coupling coordination degree between cultivated land transformation and new-type urbanization at the county level

#### Determination of impact factors

The degree of coupling coordination between the transformation of cultivated land use and new-type urbanization at the county level in the Fenhe River Basin is influenced by an array of factors including terrain, elevation, and technological advancement. Informed by previous studies^52–54^ and considering the availability of data, as well as the unique conditions of the study area, a total of 17 driving factors were selected. These factors span both natural and socio-economic dimensions and were utilized to perform single-factor analyses and multi-factor interaction assessments. The natural dimension includes four factors: temperature (*x*_1_), precipitation (*x*_2_), elevation (*x*_3_), and slope (*x*_4_). The socio-economic dimension encompasses thirteen factors: financial development (*x*_5_), fixed asset investment (*x*_6_), per capita GDP (*x*_7_), added value of the primary industry (*x*_8_), added value of the secondary industry (*x*_9_), added value of the tertiary industry (*x*_10_), population density (*x*_11_), per capita local fiscal revenue (*x*_12_), per capita local fiscal expenditure (*x*_13_), urbanization rate (*x*_14_), per capita cultivated land area (*x*_15_), grain yield per unit area (*x*_16_), and per capita total mechanical power (*x*_17_).

#### Factor analysis results

Utilizing the visual box division method, the numerical values of the detected factors were transformed into seven-level categorical variables. The geographic detector model was subsequently employed to evaluate the contribution rate of each driving factor to the spatial differentiation of the coupling coordination degree for the years 1990, 2000, 2010, and 2020. It is apparent that the spatiotemporal differentiation of the coupling coordination degree is subject to influence by multiple factors, each contributing to varying extents. As illustrated in Table 4, the added value of the tertiary industry, urbanization rate, and grain yield per unit area exhibit notably high q values, indicating strong explanatory power for the spatial differentiation of the coupling coordination degree. These factors are therefore identified as dominant explanatory variables from a spatial association perspective. Among natural factors, air temperature and elevation demonstrate limited explanatory power concerning this spatial differentiation. Over time, the q values for financial development, fixed asset investment, added value of the primary industry, and per capita total power of agricultural machinery show an increasing trend, indicating that their explanatory power for the spatial differentiation of the coupling coordination degree has grown more significant. The geographic detector results revealed that tertiary industry value, urbanization rate, and grain yield were the dominant driving factors with relatively high explanatory power. The strong influence of tertiary industry value reflects the ongoing industrial restructuring toward service‑oriented development, which reshapes land demand and urban–rural land use patterns. The high explanatory power of the urbanization rate indicates that population agglomeration and urban expansion remain fundamental forces underlying the coupled coordination dynamics. The persistent effect of grain yield highlights the constraints of food security and cultivated land protection policies throughout the study period.

**Table 4:** Detection results of driving factors for coupling coordination degree (q Value).

Index	1990	2000	2010	2020
Temperature(*x*_1_)	0.1689***	0.1667***	0.2002***	0.0874***
Precipitation(*x*_2_)	0.3119***	0.4394	0.1472***	0.1695
Elevation(*x*_3_)	0.2124***	0.1404*	0.1491***	0.0805**
Slope(*x*_4_)	0.0404	0.1134*	0.2971	0.1151*
Financial development(*x*_5_)	0.1566*	0.3849	0.2154*	0.2434
Fixed asset investment(*x*_6_)	0.0496*	0.1883***	0.1136*	0.2410***
Per capita GDP(*x*_7_)	0.4378	0.2543***	0.2533	0.3625***
Added value of the primary industry(*x*_8_)	0.0321***	0.2232***	0.4087***	0.3880***
Added value of the secondary industry (*x*_9_)	0.1627***	0.3257***	0.2156***	0.1242***
Added value of the tertiary industry(*x*_10_)	0.2251***	0.2513*	0.2469***	0.2133**
Population density (*x*_11_)	0.5069***	0.3586	0.0569***	0.1851
Per capita local fiscal revenue(*x*_12_)	0.1826*	0.1892***	0.0623**	0.0401***
Per capita local fiscal expenditure(*x*_13_)	0.3989	0.2428*	0.0813	0.0860**
Urbanization rate (*x*_14_)	0.2559***	0.4935	0.1982***	0.2002
Per capita cultivated land area (*x*_15_)	0.5244*	0.2547*	0.1117**	0.2615*
Grain yield per unit area(*x*_16_)	0.3030	0.4412	0.5227	0.3408*
Per capita total mechanical power(*x*_17_)	0.0825*	0.3202*	0.1123*	0.3608*

*** , **, and * denote statistical significance at the 1%, 5%, and 10% levels, respectively.

#### Interactive detection results

**Fig. 7:**
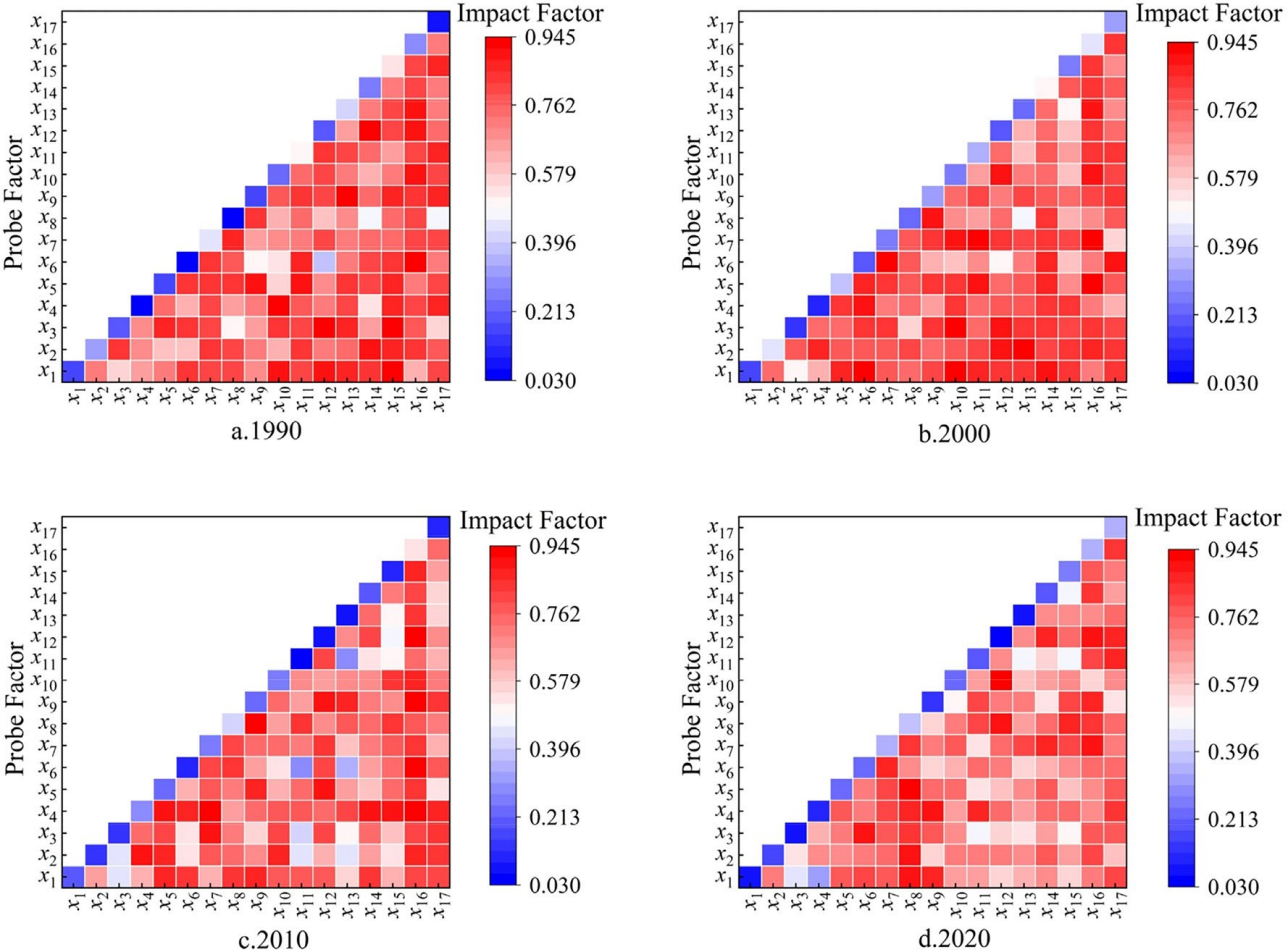
Interaction detection results for coupling coordination degree in key years.

The spatial variation in the coupling coordination degree between the transformation of cultivated land use and new-type urbanization at the county level in the Fenhe River Basin results from the synergistic influences of socioeconomic and natural factors (Fig. 7). Notably, the interplay between natural and socioeconomic factors proves more consequential than the influence of natural factors alone. Among various factors, the interactions between fixed asset investment and grain yield are particularly significant. The interaction coefficients involving temperature and the added value of the tertiary industry, fixed asset investment and per unit area grain output, the added value of the secondary industry and per unit area grain output, as well as financial development and the added value of the primary industry, all surpass 0.9. The dynamics between social and economic factors demonstrate a nonlinear interaction pattern. The degree of coupling coordination between cultivated land use transformation and new-type urbanization at the county level in the Fenhe River Basin is shaped not merely by isolated factors but through the complex interplay of multiple elements. Regarding nonlinear interactions, the combination of socioeconomic and agricultural factors exhibited significantly enhanced explanatory power, suggesting that urban industrial development and agricultural production jointly drove the spatiotemporal evolution of the coupled system, rather than acting independently.

## Discussion

### Transformation of cultivated land use

The transformation of cultivated land use involves both overt and covert changes, embodying a process that is intricate and affected by an array of factors. This process is particularly closely linked with the expansion of urban and rural construction land^55^ as well as ecological land^56^. This study develops an index system to assess the comprehensive transformation of farmland utilization in the Fenhe River Basin, incorporating aspects such as quantitative form, production function, and ecological function. Over the period from 1990 to 2020, the comprehensive index of cultivated land use transformation in the Fenhe River Basin exhibited a fluctuating yet ascending trend, increasing from 0.093 to 0.691.

In the context of transforming cultivated land use, the relationship between humans and land has undergone three distinct phases: initial deterioration, marked conflict, and gradually coordinated development. The patterns of this transformation across the upper, middle, and lower reaches of the Fenhe River Basin are notably diverse. In 2020, the middle reaches hosted 9.4073 million permanent residents, exhibited an urbanization rate of 66.31%, and generated a secondary industry output of 235.74 billion yuan, surpassing the corresponding figures in both the upper and lower reaches. This region also ranked second in terms of cultivated land area proportion, which intensified the human-land conflict. Characterized by high urbanization levels and considerable industrial concentration, the middle reaches experience more pronounced encroachment of construction land on cultivated areas. The comprehensive level of cultivated land utilization transformation in the urban area of Taiyuan, excluding the Jinyuan and Xiaodian County in the years 1990, 2000, and 2010, and the Xinghualing County in 2020, has consistently been low level. This phenomenon is primarily due to the substantial population of Taiyuan’s urban area, which restricts the per capita availability of cultivated land, thereby challenging grain security and the power of agricultural machinery. The per capita cultivated land area is a pivotal metric in understanding the shifts in land use amidst urbanization^57^. It also clarifies the interplay between agricultural labor and food production^58^, and further illuminates the evolving dynamics of human-land interactions during rapid urbanization^59^. In the dichotomous structure of urban-rural systems, a significant fraction of the rural populace migrates to urban centers, resulting in a gradual diminution of surplus rural labor. This migration leads to an initial decrease and subsequent increase in the per capita cultivated land area in rural areas, whereas urban areas witness a continual decline in the same. The low rate of cultivated land utilization and limited scale of management impede grain security per capita. However, land circulation bolsters per capita grain production, thus influencing the transformation of cultivated land use. In Shouyang County, the comprehensive level of cultivated land utilization advanced from a moderate level in 1990 and 2000 to very high level in 2010 and 2020, driven by enhancements in grain yield per unit area, per capita food security, and the total power of agricultural machinery per capita. Notably, the grain yield per unit area improved significantly over the decades, ascending from 18th place in 1990 to 4th by 2010 and maintaining this rank in 2020. Per capita food security consistently ranked among the top two positions, achieving first place by 2000. Moreover, the total power of agricultural machinery per capita saw a substantial increase, escalating from 16th place in 1990 to the leading position by 2020. Zou Baoling et al. noted that the advancement in agricultural mechanization and services significantly impacted agricultural productivity, food security, and rural household income^60^. Agricultural production conditions are continually improving, with a steady increase in the power of agricultural machinery and enhanced efficiency in cultivated land use. These improvements are actively promoting the optimization and advancement of cultivated land use transformation.

### Changes of the comprehensive level of urbanization in Fenhe River Basin

From 1990 to 2020, the Fenhe River Basin demonstrated a consistent upward trend in its comprehensive urbanization level. The average urbanization rate escalated from 14.73% in 1990 to 49.49% in 2020, mirroring the steady progress in urban development observed throughout the Yellow River Basin^61^. Due to the heterogeneous development rates across different counties, significant disparities in urbanization levels are apparent within the basin. Both population and economic urbanization^62^ exert substantial influence on the overall urbanization metrics.

In 1990, urban population percentages across counties varied from 3.46% to 42.46%, presenting a substantial range of 39 percentage points. The Gross Domestic Product (GDP) of each county ranged from 60.633 million yuan to 1,335.816 million yuan, indicating relatively modest economic disparities among the counties. The dimensions of population and economic urbanization were markedly more pronounced than those of land and social urbanization, resulting in 65.85% of counties classified under a low urbanization level, while 34.15% attained low-moderate level.

By 2000, the proportion of counties characterized by low-moderate level urbanization had escalated to 90.24%, whereas only 1% of counties were categorized as low level urbanized. The GDP figures for Lan County, Loufan County, and Fushan County were notably low at 169.11 million yuan, 220.1 million yuan, and 300.35 million yuan, respectively. These statistics positioned the counties among the lowest in GDP rankings, with Lan County being the last, Loufan County second-to-last, and Fushan County fifth from the bottom. The underdeveloped economic status of these counties^63^ directly contributed to their low urbanization levels.

In 2010, Xiaoyi City was elevated from a low-moderate level area to a high level area. Its GDP surged to 25.894 billion yuan, ranking it fifth among all counties. Rich in natural resources like coal and aluminum, the city’s secondary industry constituted 60.41% of its economy. The expansion of renewable energy resources and the growth of tertiary industries, such as finance and services, are poised to further enhance urbanization levels^64^. In Taiyuan, both Xiaodian and Jiancaoping County have experienced administrative promotions from low-moderate to high levels. Notably, Xiaodian County has witnessed continual enhancements in urban infrastructure and public services, coupled with rapid development in the tertiary sector. The proportion of tertiary industry output increased from 49.1% in 2000 to 53.8%. Since the establishment of Jiancaoping County in 1997, the urban population has grown from 202,336 to 385,135, and the real estate market demand has consistently escalated^65^, leading to a continuous expansion in urban construction land^66^ and improvements in the comprehensive urbanization level.

In 2020, there was a notable increase in the proportion of counties achieving advanced urbanization, comprising 43.9% of the total. All counties administered by Taiyuan City exhibit very high level of urbanization, with Loufan County classified as relatively high- level, spatially, these counties display a pronounced clustered distribution pattern. As the administrative hub of Shanxi Province, Taiyuan City holds significant advantages in political, economic, cultural, and policy realms. It plays a pivotal role in influencing the urbanization of neighboring counties through a radiating effect, which demonstrates a geographical pattern of attenuation. The areas with very high urbanization levels, specifically Yuci County, Taigu County, Pingyao County, Jiexiu City, and Lingshi County, are notably concentrated. Urbanization levels correlate positively with educational advancement and the presence of research institutions within universities^67^. The pronounced urbanization in Yuci County of Jinzhong City, for instance, can be attributed to several factors. These include the establishment of Shanxi University Town, an increase in the number of faculty and students, expansion of the consumer market, and growth in the surrounding commercial, real estate, and tourism sectors, all of which have accelerated the urbanization pace. Urbanization is fundamental to the development of tourism^68^ and can significantly bolster urban growth. Pingyao County, renowned for its rich tourism resources, hosted approximately 631,000 visitors in 2020, generating significant revenue from tourism tickets amounting to 15.055 million yuan. Consequently, the county’s overall urbanization level increased to 53.68%. The comprehensive urbanization levels in counties within the Fenhe River Basin are closely associated with economic development, industrialization, educational progress, population density, and tourism expansion.

### The coupling relationship between cultivated land use transformation and county-level urbanization

The interaction between the transformation of cultivated land use and county-level urbanization is mutually influential and reinforcing, establishing a complex, open, and interactive coupling relationship^69,70^. During periods of rapid urbanization, urban development often encroaches upon substantial quantities of high-quality cultivated land^71^. In response to the scarcity of arable land amidst swift urban expansion, intensive agricultural practices have been adopted to enhance yield per unit area. These practices, however, have led to the large-scale and concentrated application of fertilizers and pesticides, culminating in significant soil pollution and degradation^72^. Research by Niu Pu et al. demonstrates that urbanization propels agricultural transformation^73^, which results in a reduction in both arable land and water bodies. Similarly, Liu Miao et al. contend that urbanization, agricultural modernization, and agricultural policies are pivotal in driving the evolution and transformation of cultivated land functions^74^.

The degree of coupling coordination between farmland utilization transformation and new-type urbanization at the county level within the Fenhe River Basin is showing signs of progressive improvement, with a spatial evolution trend toward greater balance and coordination. The coupling coordination between these phenomena in 1990 and 2000 was primarily characterized by primary dissonance. This indicates that in the early stages of urbanization, the expansion of urban infrastructure and residential areas led to a significant decline in both the quantity and quality of cultivated land. The detrimental impacts on cultivated land were more pronounced than the positive effects^75^, suggesting that various levels of government paid insufficient attention to this issue. Moreover, the reciprocal nature of the relationship between urbanization and cultivated land use has been neglected, rather than being acknowledged as a unidirectional influence.

In 2010, approximately 80.49% of counties had achieved primary coordination levels of coupling coordination. By 2020, this figure had risen to 90.24%, with counties reaching intermediate or high-level coordination levels of coupling coordination. This improvement aligns with the implementation of China’s policies aimed at energy conservation and emission reduction, as well as the adoption of a green development philosophy. Consequently, the Chinese government has increasingly emphasized the impact of urbanization on agriculture and the use of cultivated land. Previously characterized by opposition and division, the relationship between urban and rural areas has evolved into one of coordination and integration^76^. This shift has facilitated the alignment of various regulations, promoting a transition from uncoordinated to coordinated land use in relation to urbanization. Shouyang County, a key area within the central Shanxi city cluster, exemplifies this progression. In 2010, the county demonstrated an intermediate coordination level of coupling coordination, with an index of 0.655. By 2020, this index had increased to 0.759, indicating a high-level of coordination. Shouyang County has actively pursued the intelligent development of its coal mines and promoted the integration of agriculture, culture, and tourism, contributing to rural revitalization. Furthermore, the county has developed new industries, such as smart logistics, intelligent manufacturing, the low-altitude economy, and biopharmaceuticals, which have enhanced the integration of farmland utilization and urbanization. In Yangqu County, Qi County, and Hejin City, a high-level coordination status in the transformation of cultivated land use and urbanization was achieved by 2020. Yangqu County has focused on developing a livable and prosperous countryside, advancing new urbanization projects, and promoting urban-rural integration. The comprehensive index of farmland utilization transformation in Yangqu County reached a notable 0.69. The investment climate has been significantly improved by the establishment of a transformation and development industrial park. By 2020, the county’s GDP had increased to 6218.86 million yuan, and the urbanization rate of permanent residents reached 53.34%, markedly enhancing the urbanization level and promoting coordinated coupling in land use transformation. In Qi County, the grain yield per unit area in 2020 was 8.6 t/hm^2^, the highest in the region. Additionally, the county ranked within the top ten for per capita cultivated land area, per capita grain supply, and per capita total power of agricultural machinery, indicating high level of comprehensive land use transformation. The land use efficiency reached 124.8 million yuan per km^2^, with the secondary industry’s added value totaling 1.989 billion yuan. These achievements have supported higher urbanization levels and enhanced the coupling coordination between farmland utilization and urbanization, resulting in a high-level of coordination.

### Limitations

Firstly, the 10-year study interval may affect the representativeness of the selected years. Future studies should consider shorter time frames to enhance the precision and applicability of the results. Secondly, this study provides a quantitative analysis of new-type urbanization at the county level in the Fenhe River Basin across four dimensions: population, economy, society, and land use. The results offer insights into the spatio-temporal differentiation of new-type urbanization at the county level, which can aid in the development of a more comprehensive new-type urbanization at the county level index system that includes ecological and regional landscape factors. Lastly, the selection of driving factors faces challenges due to data availability constraints. For natural factors, the density of the river network should be considered, while social and economic factors could be expanded to include aspects such as educational provisions, financial support for agriculture, road infrastructure, and industrial structure. The core limitation of the geodetector lies in the fact that its q value only reflects the spatial differential covariation among variables, unable to distinguish whether this covariation stems from causal effects, common driving factors, or spatial autocorrelation. Moreover, it lacks the ability to handle endogeneity and dynamic time mechanisms, and thus is essentially a tool for detecting spatial correlations rather than a causal inference method. Although widely adopted in sustainability and land-use research, the coupling coordination degree model entails notable methodological limitations. Specifically, it presumes an inherent synergistic tendency between coupled systems—a simplifying assumption that overlooks the complexity, temporal nonlinearity, and phase asynchrony characterizing the interaction between cultivated land use transformation and county-level urbanization. To address this gap, future work will integrate time-series and process-tracing dynamic analysis methods to better capture the evolving, context-dependent nature of this nexus.

## Conclusions

This study, which centers on the Fenhe River Basin and specifically examines the years 1990, 2000, 2010, and 2020, employs the coupling coordination degree model and kernel density analysis to explore the dynamic relationship and influencing factors between cultivated land use transformation and county-level urbanization. The findings are summarized below:

- (1) Cultivated Land Use Transformation: The comprehensive level of cultivated land use transformation in the Fenhe River Basin exhibited an upward trend during the study period, with the proportion of counties reaching high transformation levels increasing from 9.3% in 1990 to 69.09% in 2020, a rise of 59.79 percentage points. Spatially, counties with high transformation levels expanded notably in the middle and lower reaches of the basin.
- (2) New-type Urbanization at the County Level: The comprehensive level of county-level urbanization in the Fenhe River Basin exhibited a consistent upward trend from 1990 to 2020. The proportion of counties with low-to-moderate urbanization levels decreased from 34.15% to 2.43%, while the share of counties with very high urbanization levels increased to 43.90%. Spatially, very high-level urbanization zones were concentrated in the Taiyuan urban area and its surroundings, including Xiaoyi City and Yaodu County.
- (3) Coupling Coordination: The overall coupling coordination between cultivated land use transformation and new-type urbanization at the county level in the Fenhe River Basin increased progressively from 1990 to 2020. The share of counties with primary coordination rose from 2.43% in 1990 to 80.49% in 2010, while the share with intermediate coordination increased from 4.89% in 2010 to 63.41% in 2020. Spatially, counties with high-level coordination remained limited and exhibited a scattered distribution pattern.
- (4) Influencing Factors: The spatial differentiation of coupling coordination between cultivated land use transformation and new-type urbanization at the county level showed varying degrees of association with natural, social, and economic factors. Among these, the added value of the primary industry, urbanization rate, financial development, and per capita GDP exhibited relatively high explanatory power for spatial variation, whereas temperature and elevation showed relatively low explanatory power.

## Supplementary Information

Supplementary Information 1.

Supplementary Information 2.

Supplementary Information 3.

## Data Availability

The original contributions presented in the study are included in the article material, further inquiries can be directed to the corresponding author.
